# Autophagy-related genes are induced by histone deacetylase inhibitor suberoylanilide hydroxamic acid via the activation of cathepsin B in human breast cancer cells

**DOI:** 10.18632/oncotarget.18410

**Published:** 2017-06-08

**Authors:** Han Han, Jing Li, Xiuyan Feng, Hui Zhou, Shanchun Guo, Weiqiang Zhou

**Affiliations:** ^1^ Key Laboratory of Environmental Pollution and Microecology of Liaoning Province, Shenyang Medical College, Huanggu, Shenyang City, Liaoning Province 110034, P. R. China; ^2^ Department of Biochemistry and Molecular Biology, Shenyang Medical College, Huanggu, Shenyang City, Liaoning Province 110034, P. R. China; ^3^ The Second Affiliated Hospital of Shenyang Medical College, Heping, Shenyang City, Liaoning Province 110002, P. R. China; ^4^ RCMI Cancer Research Center, Xavier University of Louisiana, New Orleans, LA 70125, USA; ^5^ Department of Chemistry, Xavier University of Louisiana, New Orleans, LA 70125, USA

**Keywords:** SAHA, autophagy, apoptosis, cathepsin B, cell cycle

## Abstract

Autophagy is involved in modulating tumor cell motility and invasion, resistance to epithelial-to-mesenchymal transition, anoikis, and escape from immune surveillance. We have previous shown that SAHA is capable to induce several apoptosis and autophagy-related gene expression in breast cancers. However, the exact mechanisms of autophagy activation in this context have not been fully identified. Our results showed that the expression and the activity of Cathepsin B (CTSB), one of the major lysosomal cysteine proteases, were significantly increased in MDA-MB- 231 and MCF-7 cells upon SAHA treatment. We confirmed that Cystatin C, a protease inhibitor, significantly inhibited the expression of CTSB induced by SAHA on breast cancer cells. We demonstrated that SAHA is able to promote the expression of LC3II, a key member in the maturation of the autophagosome, the central organelle of autophagy in breast cancer cells. However, SAHA induced LC3II expression is effectively suppressed after the addition of Cystatin C to the cell culture. In addition, we identified a number of genes, as well as the mitogen-activated protein kinase (MAPK) signaling that is potentially involved in the action of SAHA and CTSB in the breast cancer cells. Overall, our results revealed that the autophagy-related genes are induced by SAHA via the activation of CTSB in breast cancer cells. An improved understanding of SAHA molecular mechanisms in breast cancer may facilitate SAHA clinical use and the selection of suitable combinations.

## INTRODUCTION

Breast cancer remains as the most common malignant disease in women in the world [1]. Although multimodality treatment strategies have been proposed for eradicating breast cancer, the incidences of breast cancer have showed a sustained upward trend for many breast cancer patients, especially estrogen receptor (ER)-negative seriously threatening their health and quality of life. Patients with ER-negative breast cancer often present high degrees of malignancy, aggression, and poor prognosis despite initial responsiveness to chemotherapy [2–3].

Epigenetic processes are direct heritable changes in gene expression without involving direct changes to the DNA sequences and play an important role in carcinogenesis [4–6]. Both the active and silent epigenetic genes are controlled by the processes of addition or removal of chemical modifications in the chromatin. These modifications include a variety of post-translational histone modifications (acetylation, phosphorylation, etc.). In recent years, epigenetic genes have been reported to be acetylated in breast cancer cell lines or breast tumors and most of them play critical roles in cell-cycle progression, differentiation, apoptosis, and autophagy [7–11].

Suberoylanilide hydroxamic acid (SAHA, vorinostat) inhibits histone deacetylase (HDAC) activity by acting on all 11 known human class I and class II HDACs, is considered the one of the most studied pan HDAC inhibitor [12]. SAHA causes growth arrest and death in a broad variety of tumors and is approved for clinical treatments of T-cell lymphoma [12–13]. A number of studies, including ours, have demonstrated that SAHA can also be effective in the inhibition of proliferation and progression in breast cancer cell lines and in animal tumor models [14–19]. Recent studies also demonstrated that SAHA combined with ionizing radiation or glucose- 6-phosphate dehydrogenase inhibitor could serve as potential therapeutic strategies for breast cancer [20–21]. However, SAHA has a short half-life of 2 hrs, due to rapid hepatic glucuronidation, making it difficult to provide the level of drug exposure necessary for durable therapeutic efficacy on solid tumors. In addition, SAHA has been ineffective against solid tumors in many clinical trials and oncogenic K-ras may contribute to SAHA resistance by upregulating HDAC6 and c-myc expression in cancer cells [22].

Cysteine cathepsins, a family of eleven human cysteine proteases that is originally characterized as main players in protein turnover within lysosomes, are highly upregulated in a wide variety of tumors by mechanisms ranging from gene amplification to post-transcriptional modification [23–24]. Cathepsin B (CTSB) is one of the major lysosomal cysteine proteases that functions in protein degradation of extracellular matrix proteins, a process promoting invasion, metastasis of tumor cells and tumor angiogenesis, and high levels of CTSB are found in a wide variety of human cancers including breast cancer [25–26]. Targeting CTSB alone does not appear to abolish tumor growth, and this is probably because CTSB appears to have diverse functions and influence numerous pathways [27]. An increase in the CTSB enzymatic activity in tumor cells treated with SAHA or other HDAC inhibitors has been reported, typically in association with apoptotic programmed cell death and autophagy [28–29]. However, a number of clinical reports have shown that CTSB was overexpressed and localized to the invasive breast tumor margin, correlating with higher aggression and poorer prognosis [30–32]. It is implied that there has a balance between breast cancer invasion and death, possibly involving in CTSB regulation. Cystatin C, a CTSB inhibitor (CBi), was also detected in breast cancer cells and its interaction with CTSB may play an important role in breast cancer invasion and metastasis [33–36].

Autophagy, a lysosomal degradation process, has been shown to be involved in modulating tumor cell motility and invasion, resistance to epithelial-to-mesenchymal transition, anoikis, and escape from immune surveillance, with emerging functions in establishing the pre-metastatic niche and other aspects of metastasis [37–39]. We have demonstrated that SAHA is capable of inducing several apoptosis and autophagy-related genes expression associated with the increased expression of CTSB in breast cancer MDA-MB-231 and MCF-7 cells [19]. Some studies have also shown that SAHA induces autophagy and exhibits potent anti-proliferative activity in breast cancer cells [40–42]. CTSB acts as a cysteine protease that is predominantly present in lysosomes and has hydrolytic enzyme activity and endopeptidase activity. When a large number of CTSB extravasation in lysosomes exceeds the conventional metabolic requirement of cancer cells, CTSB triggers a series of biological effects, including cell autophagy [43]. There is an important correlation between CTSB and SAHA-induced breast cancer cell autophagy. SAHA is able to induce caspase-independent autophagic cell death rather than apoptotic cell death in tamoxifen-resistant human breast cancer cells, thus SAHA-mediated autophagic cell death is a promising new strategy for the patients with tamoxifen-resistant breast cancer [44]. However, the exact mechanisms of autophagy activation in this context have not been fully identified. In light of the inferred associations between SAHA-induced autophagy and CTSB activity, we hypothesized that SAHA-CTSB may activate molecular mechanisms conducive to the autophagy in breast cancer cells. The aim of the present study is to investigate whether or not the autophagy-related genes are induced by SAHA via the activation of CTSB in breast cancer cells.

## RESULTS

### The effect of SAHA/Cystatin C combination on the expression and the activity of CTSB

**Figure 1:**
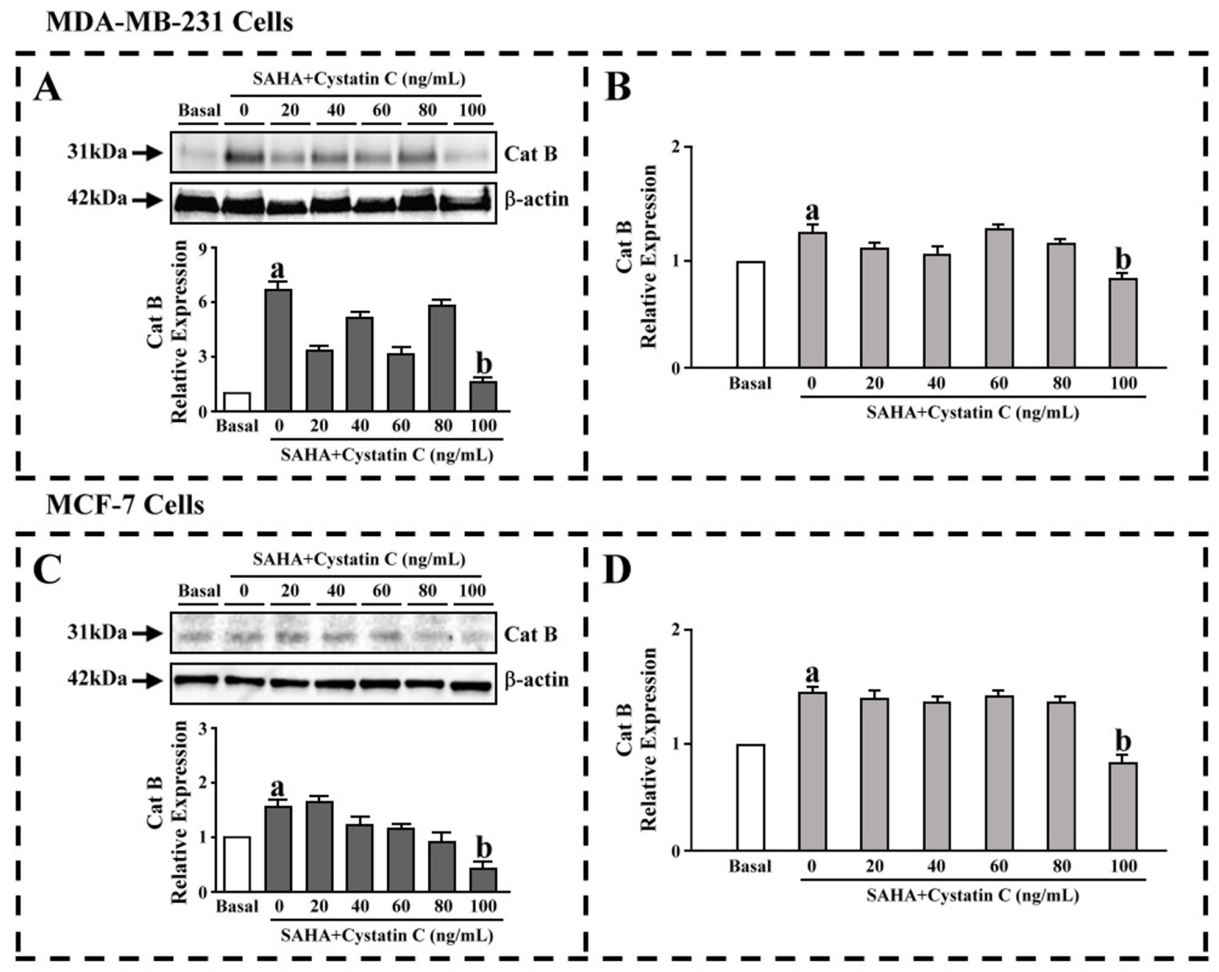
The effect of SAHA/Cystatin C combination on CTSB MDA-MB-231 or MCF-7 cells were co-cultured in the presence of Cystatin C at 0–100 ng/ml of varying concentrations. **(A)** The CTSB levels in MDA-MB-231 cells. **(B)** The activity of CTSB in MDA-MB-231 cells. **(C)** The CTSB levels in MCF-7 cells. **(D)** The activity of CTSB in MCF-7 cells. (a) *p* <0.05, (b) *p* <0.01, when comparing to basal. Data (mean ±standard error) representative results derived from a minimum of 3 independent experiments.

Preliminary experiments were performed to evaluate the effects exerted by the SAHA/Cystatin C combination in MDA-MB-231 and MCF-7 cells. We first determined the expression of CTSB upon the drug treatment by western blot assay. MDA-MB-231 or MCF-7 cells were co-cultured in the presence of Cystatin C at 0–100 ng/ ml of varying concentrations and SAHA, respectively. The results revealed that the expression of CTSB were significantly increased in MDA-MB- 231 and MCF-7 cells when Cystatin C was 0 ng/ml, which indicated that SAHA increased the expression of CTSB. After quantitation of the integrated intensity of the images by ImageJ software, the CTSB levels increased by 6.5- folds in MDA-MB-231 cells and 1.5- folds in MCF-7 cells, respectively (Figure 1A,C, lower panels). It is noted that Cystatin C at 100 ng/ml concentration, the CTSB levels were significantly lower in both MDA- MB-231 and MCF-7 cells. In contrast to MDA-MB- 231 cells, the CTSB expression in MCF-7 cells showed apparent dose-dependency upon SAHA/Cystatin C treatment (Figure 1A,C).

ELISA was then used to further evaluate the activity of CTSB. Similar to the expression of CTSB, the activities of CTSB were significantly increased in MDA-MB-231 and MCF-7 cells when Cystatin C was 0 ng/ml. The activities of CTSB levels were also significantly decreased in both MDA-MB-231 and MCF-7 cells once 100 ng/ml of Cystatin C was added (Figure 1B,D).

### The effect of SAHA/Cystatin C combination on CTSB

**Figure 2:**
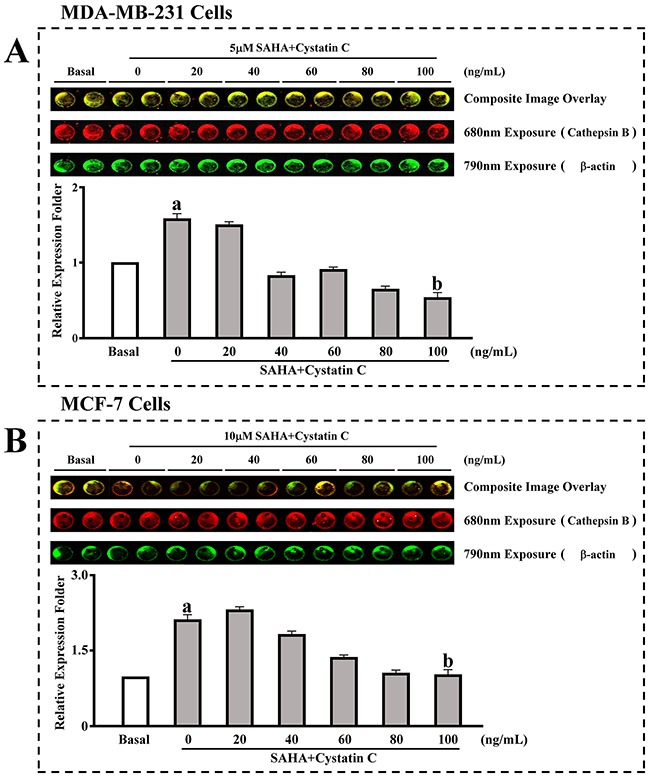
In cell western assay for the effect of SAHA/Cystatin C combination on CTSB MDA-MB-231 or MCF-7 cells were incubated with 5 μM, 10 μM and different concentrations of Cystatin C. **(A)** The expression of CTSB in MDA-MB-231cells. **(B)** The expression of CTSB in MCF-7 cells. (a) *p* <0.05, (b) *p* <0.01, when comparing to basal. Data (mean ±standard error) representative results derived from a minimum of 3 independent experiments.

We then confirmed the above results using a in cell western assay. MDA-MB-231 or MCF-7 cells were incubated with SAHA (5-10 μM) and different concentrations of Cystatin C (0, 20, 40, 60, 80 and 100ng/ml). We found that in the group with SAHA treatment, the expression of CTSB was significantly increased in both cell lines (Figure 2A,B). The CTSB levels were increased by 1.6- folds in MDA-MB-231 cells and by 2.1- folds in MCF-7 cells. With the increased concentration of Cystatin C, the expression of CTSB was decreased. With Cystatin C at 100 ng/ml, the levels of CTSB that reached the minimum were significantly decreased in its expression compared to SAHA treatment in both MDA- MB-231 and MCF-7 cells.

### The effect of SAHA/Cystatin C combination on the cell viability and apoptosis

**Figure 3:**
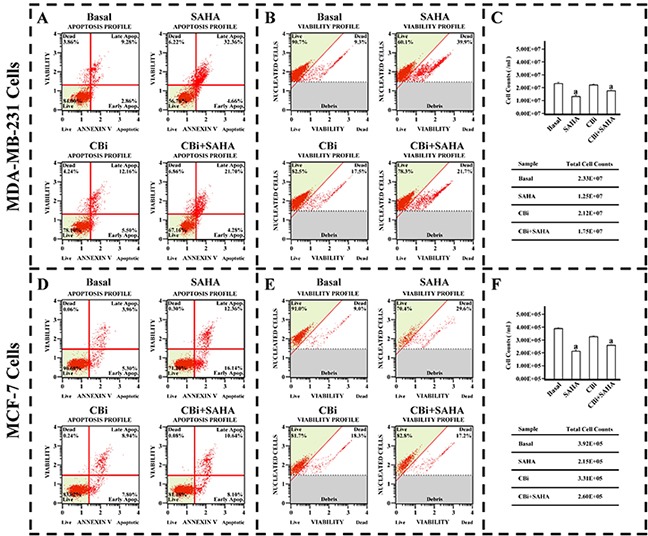
The effect of SAHA/Cystatin C combination on cell viability and apoptosis of cancer cells MDA-MB-231 or MCF-7 cells were plated in 6-well plate. 5μM SAHA and 100 ng/ml Cystatin C in treatment of MDA-MB-231 cells. 10μM SAHA and 100 ng/ml Cystatin C in treatment of MCF-7 cells. **(A)** The apoptosis profile in MDA-MB-231. **(B)** The cell viability profile in MDA-MB-231. **(C)** The cell number in MDA-MB-231. **(D)** The apoptosis profile in MCF-7. **(E)** The cell viability profile in MCF-7. **(F)** The cell number in MCF-7. (a) *p* <0.05, when comparing to basal. Data (mean ±standard error) representative results derived from a minimum of 3 independent experiments.

In order to investigate the effects of SAHA and Cystatin C on breast cancer cell proliferation, we determined the cell viability and apoptosis in MDA-MB-231 and MCF-7 cell lines. In comparison with DMSO control treatment, both cell viability and cell number decreased in MDA-MB-231 and MCF-7 cells after SAHA treatments. While there was no significant difference between DMSO and CBi in inhibiting growth of both cell lines, the combination of CBi and SAHA treatment induced dramatic decreases in cell viability and cell number of both MDA-MB-231 and MCF-7 cells. (Figure 3B,C,E,F). As expected, in comparison with DMSO control treatments, the apoptotic cells increased in MDA-MB-231 and MCF-7 cells after the SAHA treatment. CBi alone only showed slight increase in apoptotic cells in the two cell lines. However, the apoptotic cells dramatically increased in MDA-MB-231 and MCF-7 cells after combining CBi and SAHA treatment; the apoptotic rate reached 4.28% in the early stage and 21.70% in the late stage in MDA-MB-231. The apoptotic rate reached 8.10% in the early stage and 10.64% in the late stage in MCF-7 cells (Figure 3A,D).

### The effect of SAHA/Cystatin C combination on LC3II

**Figure 4:**
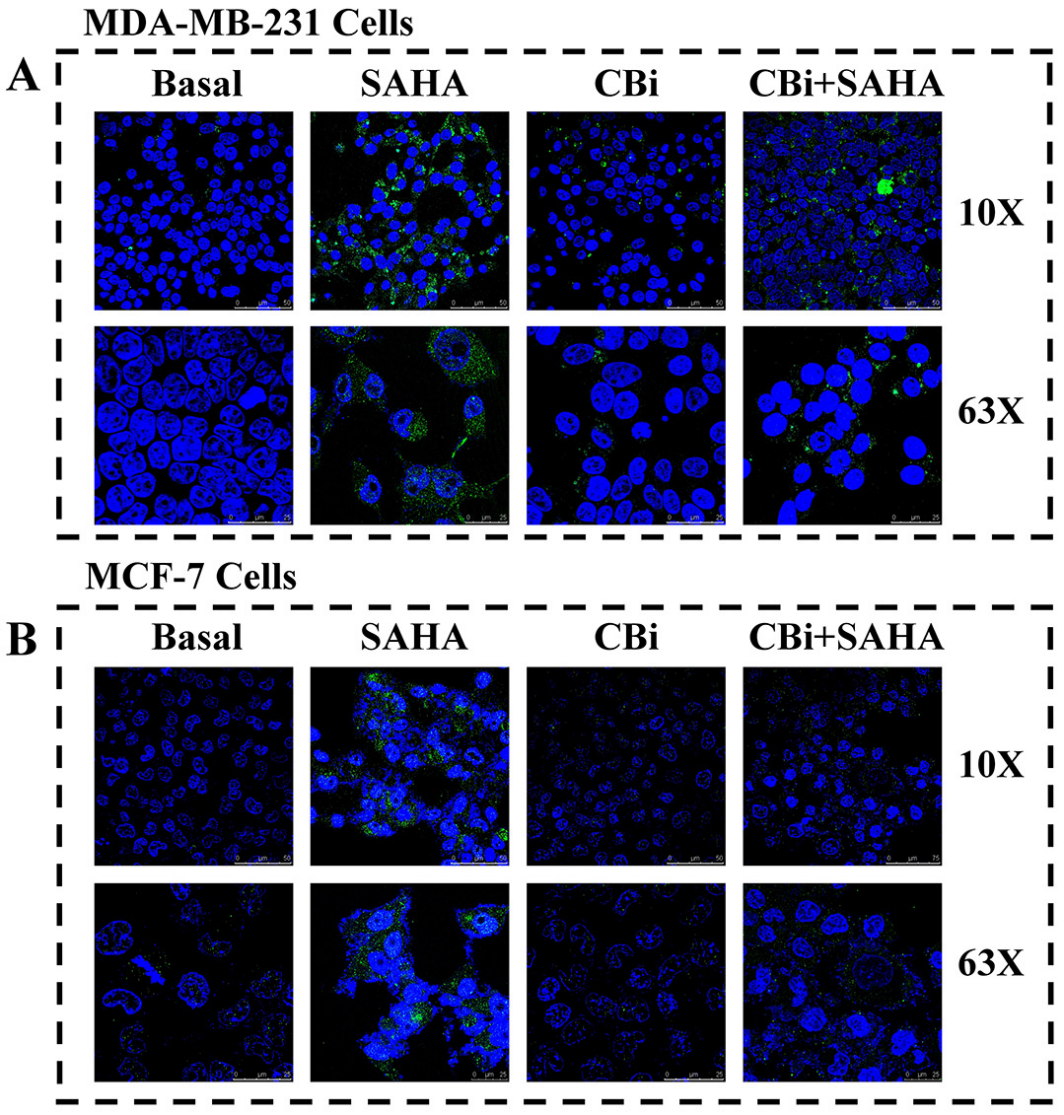
The effect of SAHA/Cystatin C combination on LC3II MDA-MB-231 or MCF-7 cells were incubated with SAHA and Cystatin C in 6-well plate. 5μM SAHA and 100 ng/ml Cystatin C in treatment of MDA-MB-231 cells. 10μM SAHA and 100 ng/ml Cystatin C in treatment of MCF-7 cells. **(A)** The fluorescence signal of LC3II in MDA-MB-231. **(B)** The fluorescence signal of LC3II in MCF-7.

In order to clarify the effects of autophagy by SAHA and Cystatin C in breast cancer cells, we determined LC3II by fluorescence microscopy. The cells grew well and occupied 90% of the full plate in the control treatment of DMSO, without LC3II. On the contrary, there was a large number of LC3II in the cytoplasm of both MDA-MB-231 and MCF-7 cells after SAHA treatment. We observed distinct changes in cancer cells after CBi treatment alone compared to in combined SAHA treatment. For MDA-MB-231 cells, there was a little LC3II after CBi treatment alone, and some LC3II after CBi plus SAHA treatment. For MCF-7 cells, there was no LC3II in the cytoplasm (Figure 4).

### The effect of SAHA/Cystatin C combination on the expression of autophagy-related molecules in cancer cells

**Figure 5:**
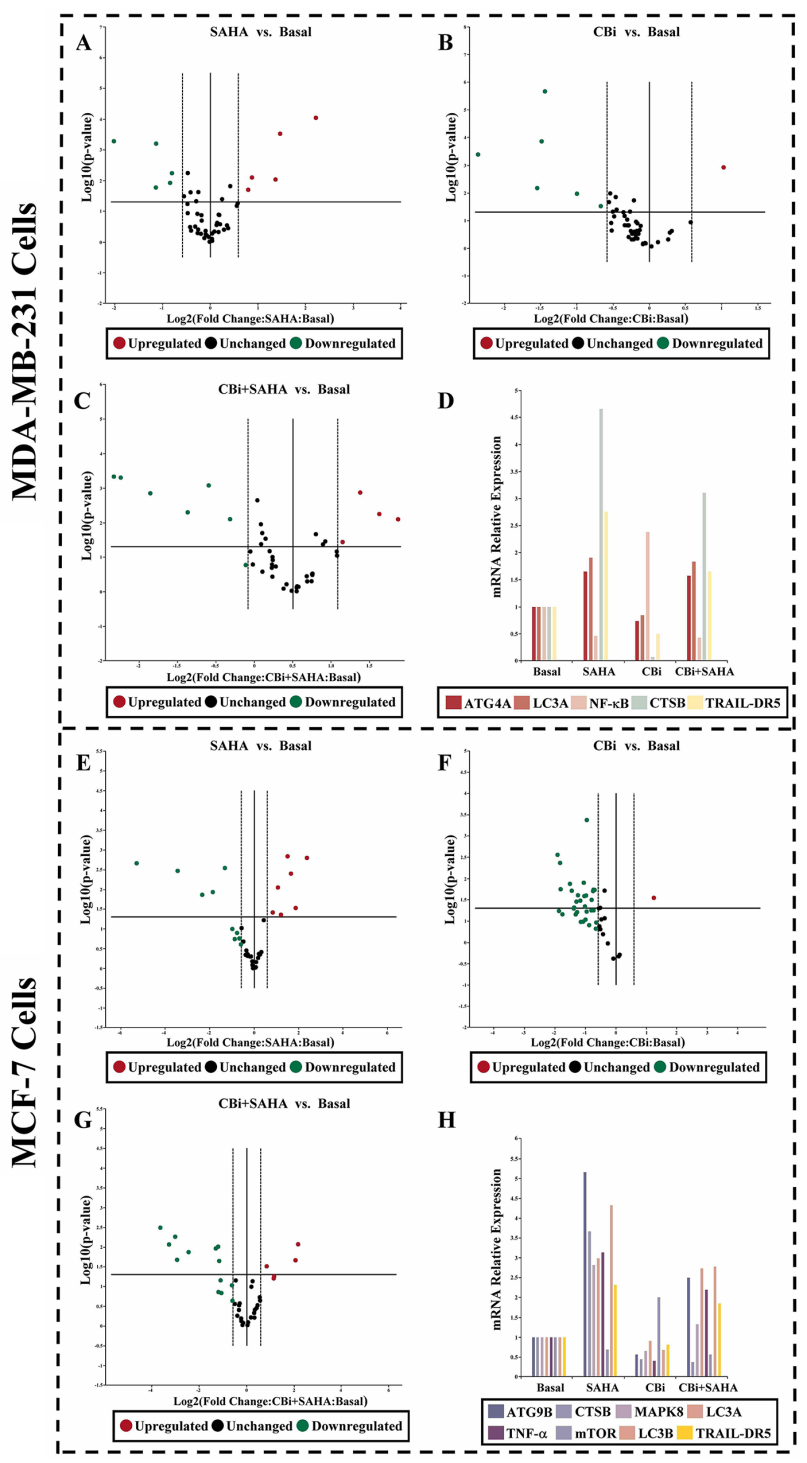
The effect of SAHA/Cystatin C combination on the expression of autophagy-related molecules in cancer cells 5μM SAHA and 100 ng/ml Cystatin C in treatment of MDA-MB-231 cells. 10μM SAHA and 100 ng/ml Cystatin C in treatment of MCF-7 cells. Applied Biosystems 7500 real-time PCR system following manufacturer’s instructions. **(A-D)** The identified genes in MDA-MB-231 cells. **(E-H)** The identified genes in MCF-7 cells. (a) *p* <0.05, (b) *p* <0.01, when comparing to basal. Data (mean ±standard error) representative results derived from a minimum of 3 independent experiments.

To investigate the potential involvement of signaling mechanisms in proliferation and inhibition, as well as autophagy induction by SAHA and CTSB for breast cancer cells. We used real-time PCR arrays to determine the expression levels of the genes. We found that, the trend of CBi and SAHA treatment combination is similar to SAHA alone, which is opposite to CBi alone. For MDA-MB-231 cells, there were 4 genes upregulated and 1 gene downregulated after the treatment with CBi and SAHA, LC3A level was increased by 3.1-folds. For MCF-7 cells, there were 6 genes upregulated and 2 genes downregulated after treatment with CBi and SAHA, ATG9B, LC3A and LC3B levels were increased by over 2.5-folds (Figure 5).

**Figure 6:**
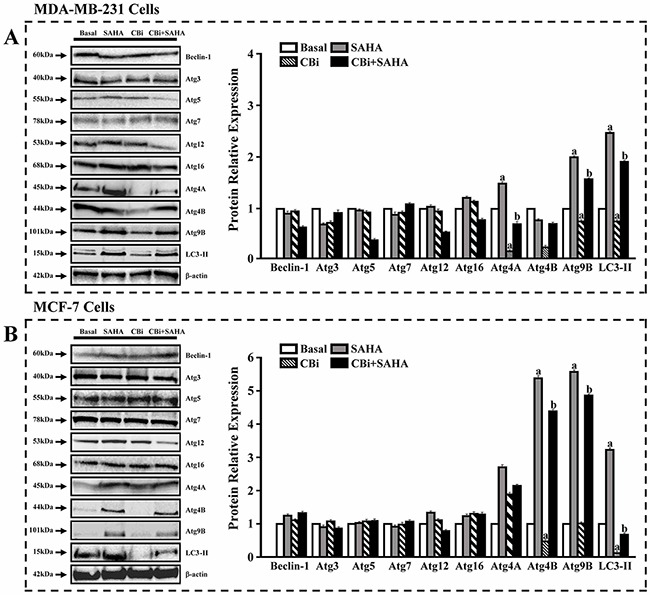
The effects of SAHA and Cystatin C on the expression of ATGs and LC3. Western blot analysis 5μM SAHA and 100 ng/ml Cystatin C in treatment of MDA-MB-231 cells. 10μM SAHA and 100 ng/ml Cystatin C in treatment of MCF-7 cells. **(A)** A number of proteins were validated in MDA-MB-231. **(B)** A number of proteins were validated in MCF-7. (a) *p* <0.05, (b) *p* <0.01, when comparing to basal. Data (mean ±standard error) representative results derived from a minimum of 3 independent experiments.

To further confirm the effects of SAHA and CTSB on autophagy, we used western blot to examine the expression of ATGs and LC3. For MDA-MB-231 cells, SAHA only significantly increased ATG4A, ATG4B, ATG9B and LC3II. CBi only significantly decreased ATG4A and ATG4B. SAHA and CBi significantly increased ATG9B and LC3II. For MCF-7 cells, SAHA only significantly increased ATG4A, ATG9B and LC3II. CBi only significantly decreased ATG4A and LC3II. SAHA and CBi significantly increased ATG4A and ATG9B (Figure 6).

### The effect of SAHA/Cystatin C combination on the MAPK signaling

**Figure 7:**
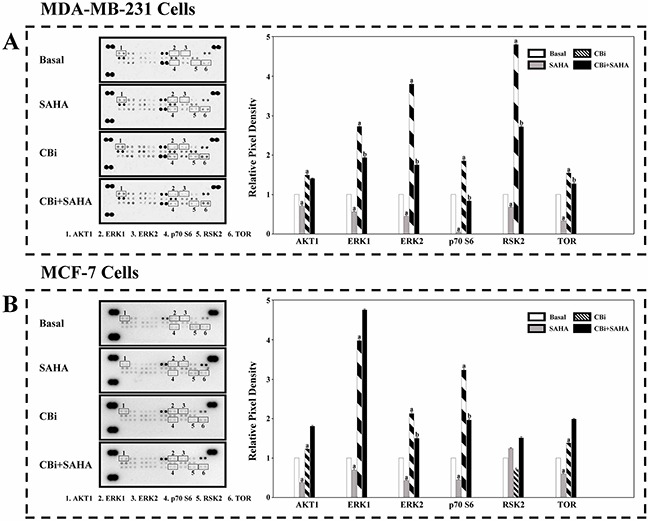
The effect of SAHA/Cystatin C combination on the MAPK signaling mechanisms in cancer cells An antibody array. 5μM SAHA and 100 ng/ml Cystatin C in treatment of MDA-MB-231 cells. 10μM SAHA and 100 ng/ml Cystatin C in treatment of MCF-7 cells. **(A)** A number of members in MAPK signaling were identified and their relative expression in MDA-MB-231 cells. **(B)** A number of members in MAPK signaling were identified and their relative expression in MCF-7 cells. (a) *p* <0.05, (b) *p* <0.01, when comparing to basal. Data (mean ±standard error) representative results derived from a minimum of 3 independent experiments.

In order to clarify the MAPK signaling mechanisms induced by SAHA and CTSB, we determined a number of protein expression using an antibody array. We found that the trend of CBi and SAHA treatment is similar to CBi alone, which is opposite to SAHA alone. For MDA-MB-231 cells, SAHA only significantly decreased ERK1, ERK2, p70S6 and TOR. CBi only significantly increased AKT1, ERK1, ERK2, p70S6, RSK2 and TOR. SAHA and CBi significantly increased ERK1, ERK2, RSK2 and TOR. For MCF-7 cells, SAHA only significantly decreased AKT1, ERK1, ERK2, p70S6 and TOR. CBi only significantly increased ERK1, ERK2, p70S6 and TOR. SAHA and CBi significantly increased AKT1, ERK1, ERK2, p70S6, RSK2 and TOR (Figure 7).

## DISCUSSION

We have demonstrated in previous report that SAHA is able to induce growth arrest and promoted apoptosis in breast cancer cells [19]. CTSB has a prominent function in mediating apoptosis potentiated by sodium butyrate (an HDACi), and doxorubicin combinations in myeloma [29]. In order to explore whether or not CTSB is also involved in the action of SAHA on breast cancer cells, we used western blot and ELISA assay to determine CTSB protein levels in breast cancer cells upon SAHA treatment. Through our observation, we can see clearly that SAHA indeed is able to induce protein level expression of CTSB. This phenomenon indicates that CTSB is potentially involved in the action of SAHA on breast cancer cells. Next, we applied different concentrations of Cystatin C [45–46], a protease inhibitor and SAHA on MDA-MB-231, MCF-7 cells. We found that large doses of Cystatin C (100ng / ml) were significantly inhibiting the expression of CTSB induced by SAHA. The overall level of protein expression, as shown through in cell western analysis, also confirms our observation.

To further confirm the effects of SAHA induced CTSB on breast cancer cells, we determined the apoptosis, cell viability and cell growth in MDA-MB-231 and MCF-7 cell lines by using Muse flow cytometry. As can be seen from Figure 3, SAHA can significantly induce apoptosis in both MDA-MB-231 and MCF-7 cells. Cystatin C alone had minimal effects on the MDA-MB-231 and MCF-7 cells; however, the combination of Cystatin C and SAHA significantly reversed the inhibitory effect of SAHA on the growth of breast cancer cells. These results indicate that CTSB plays a significant role in regulating the action of SAHA in the inhibition of breast cancer cell growth and promotion of apoptosis.

Members of the LC3 family play a key role in the maturation of the autophagosome, the central organelle of autophagy [47–49]. LC3 precursors are proteolytically processed to form LC3I, which is distributed in the cytosol through diffusion. Upon the initiation of autophagy, the C-terminal glycine of LC3I is modified by the addition of a phosphatidylethanolamine (PE) to form LC3II, which translocates rapidly to nascent autophagosomes in a punctate distribution. In order to investigate the impacts of SAHA induced CTSB on autophagy in breast cancer cells, we checked LC3II in the cells treated with SAHA and Cystain C using laser scanning confocal microscopy. Our results showed that the fluorescence signal of LC3II significantly increased, indicating that there are a large number of LC3II in the cytoplasm of both MDA-MB-231 and MCF-7 cells after SAHA treatment. However, there were a little LC3II after CBi treatment alone, and some LC3II after CBi plus SAHA treatment in MDA-MB-231 cells. For MCF-7 cells, there was no LC3II in the cytoplasm after CBi treatment alone or CBi plus SAHA treatment. These results indicate that SAHA can promote the expression of LC3II in breast cancer cells. However, SAHA induced autophagy is effectively suppressed, after the addition of Cystatin C to the cell culture, since the function of CTSB is effectively suppressed by Cystatin C, and LC3II expression significantly decreased.

To further explore the molecular mechanisms in the action of SAHA and CTSB in breast cancer cells, we used real-time PCR arrays to determine the expression levels of the genes. We identified 4 genes upregulated and 1 gene downregulated in MDA-MB-231cells after treatment with CBi and SAHA. We also identified 6 genes upregulated and 2 genes downregulated in MCF-7 cells after treatment with CBi and SAHA. SAHA and CBi significantly increased ATG9B and LC3II using western blot analysis in MDA-MB-231 cells. SAHA and CBi also significantly increased ATG4A and ATG9B as indicated by the western blot analysis of MCF-7 cells.

Lastly, we clarified that the mitogen-activated protein kinase (MAPK) signaling may involve in the action of SAHA and CTSB in the breast cancer cells. MAPKs are key regulators of cell growth and survival in physiological and pathological processes [50–51]. Aberrant MAPK signaling plays a critical role in the development and progression of human cancer including breast cancer, as well as in determining responses to cancer treatment [52–54]. We identified that SAHA and CBi significantly increased ERK1, ERK2, RSK2 and TOR in MDA-MB-231 cells. In contrast, SAHA and CBi significantly increased AKT1, ERK1, ERK2, p70S6, RSK2 and TOR in MCF-7 cells. The finding that SAHA and CBi are able to activate certain MAPK signaling pathways in breast cancer cells may have significant clinical values. Currently, a number of MAPK inhibitors have been developed and further explored in clinical trials of cancer patients [55–57]. The effects of SAHA combined with MAPK inhibitors need for further investigation in breast cancer.

In conclusion, our results showed that the expression and the activity of CTSB were significantly increased in MDA-MB- 231 and MCF-7 cells upon SAHA treatment. Cystatin C, a protease inhibitor, significantly inhibited the expression of CTSB induced by SAHA on breast cancer cells. We demonstrated that SAHA is able to promote the expression of LC3II, which effectively suppressed, with the addition of Cystatin C to the cell culture. Additionally, we identified a number of genes, as well as MAPK signaling that is potentially involved in the action of SAHA and CTSB in the breast cancer cells. Overall, our results revealed that the autophagy-related genes are induced by SAHA via the activation of CTSB in breast cancer cells. An improved understanding of the molecular mechanisms of SAHA action in breast cancer may facilitate SAHA clinical applications and the selection of suitable combinations.

## MATERIALS AND METHODS

### Cell lines and reagents

Human MDA-MB-231 and MCF-7 cells were purchased from American Type Culture Collection (ATCC) (Manassas, VA). Leibovitz’s L-15 medium, RPMI-1640 medium, Fetal Bovine Serum (FBS), Penicillin-streptomycin Cocktails and Cystatin C (Catalog number: PHP0044) were obtained from Thermo Scientific (Rockford, IL). SAHA was purchased from Sigma-Aldrich (St. Louis, MO). Muse Cell Cycle kit, Muse Annexin & Dead Cell kit, and Muse Count & Viability kit were from Millipore (Darmstadt, Germany). Human MAPK Antibody Array kit was purchased from R&D Systems (Minneapolis, MN). High Pure RNA Isolation kit and Transcriptor First Strand cDNA Synthesis kit were obtained from Roche Diagnostics GmbH (Mannheim, Germany). Exprofile Human autophagy Gene qPCR Array kit was obtained from Genecopoeia (Rockville, MD). Power SYBR Green PCR Master mix, RIPA Cell Lysis buffer and BCA Protein Assay kit were from Life Technologies (Austin, TX). Polyclonal anti-beclin-1 antibody, polyclonal anti-Atg3 antibody, polyclonal anti-Atg5 antibody, polyclonal anti-Atg7 antibody, polyclonal anti-Atg12 antibody, polyclonal anti-Atg16 antibody, polyclonal anti-Atg4A antibody, polyclonal anti-Atg4B antibody, polyclonal anti-Atg9B antibody, polyclonal anti-LC3II antibody were obtained from Abcam Inc (Cambridge, MA). Protease inhibitor and other chemicals were purchased from Sigma-Aldrich (St. Louis, MO).

### Cell culture

MDA-MB-231 or MCF-7 cells were grown in Leibovitz’s L-15 medium or RPMI-1640 medium, respectively, with 15% fetal bovine serum (FBS), 100U/ml penicillin and 100μg/ml streptomycin. The cells were incubated in a 95% humidified atmosphere at 37°C with 5% CO_2_. Cells were seeded in 96-wells plate (1.0×10^4^ cells/ml), 6-wells plate (5.0×10^5^ cells/ml) and 100mm dish (1.5×10^7^ cells/ml). Semi-confluent cells were starved for 24 hours in basal medium (with DMSO) without FBS and treated with different compounds.

### Cystatin C dose-response effects

MDA-MB-231 or MCF-7 were starved as described above and incubated for 24 hours with medium containing Cystatin C (0, 20, 40, 60, 80 and 100ng/ml). Cellular lysate were used to assess Cystatin C dose-response effects using western blot, ELISA and in cell western according to the manufacturer protocol.

### Cell viability, apoptosis and cell cycle assay

For MDA-MB-231, cells were plated in 6-well plate. After synchronization with 5μM DMSO (basal medium) without FBS for 24 hours, the cells were incubated in complete culture medium containing 5μM SAHA or combining with 100ng/ml Cystatin C for 48 hours. For MCF-7, cells were also plated in 6-well plate. After synchronization, the cells were incubated in complete culture medium containing 10μM SAHA or combining with 100ng/ml Cystatin C for 24 hours.

Muse Count & Viability reagent was used to assess the cell viability. 2×10^5^ of harvested cells (50ul cell suspension) was added with 450ul Count & Viability reagent. The results were obtained with Muse Count & Viability software module, and the statistics showed the concentration and percentage of viable cells.

For the apoptotic assay, 1×10^6^ of cells were incubated with 100μl of Muse Annexin V & Dead Cell reagent for 20 minutes at room temperature. Muse Cell Analyzer determined apoptosis, and the statistics showed the percentages of the cells represented by alive, apoptosis and dead population.

### RNA extraction and real-time PCR array

RNA was extracted from MDA-MB-231 or MCF-7 cells using High Pure RNA Isolation kit. First-strand cDNA, synthesized from total RNA and cDNA, was used as a template in real-time PCR reactions with Power SYBR Green PCR Master mix and was run on an Applied Biosystems 7500 real-time PCR system following the manufacturer’s instructions. The 25μl real-time quantitative PCR reaction mixture consisted of 1x SYBR Green Supermix and 10ng cDNA. Data normalization was based on correcting all *C*_t_ values for the average *C*_t_ values of GAPDH gene present on the array. Three independent biological replicates were performed. Quantitative PCR-array data was analyzed following the reported procedures.

### Western blot analysis

MDA-MB-231 or MCF-7 cells were washed once with ice-cold PBS and disrupted by homogenization in RIPA cell lysis buffer contained 0.1mg/ml protease inhibitor, 1mM PMSF. Cellular lysate was rotated for 2 hours at 4°C followed by centrifugation for 10 minutes at 14,000g at 4°C. Proteins were quantified using BCA protein assay kit. 20μg proteins were loaded per lane on SDS-polyacrylamide gels and transferred to PVDF membranes. Western blot analyses were performed using the antibodies described above. Level of β-actin was used as loading controls. Protein-bands were detected using ECL Western blot substrate and exposed on DNR MF-Chemi Bio-Imaging Systems.

### In cell western assay

MDA-MB-231 or MCF-7 cells were incubated with SAHA and different concentrations of Cystatin C (0, 20, 40, 60, 80 and 100ng/ml) in a 96-well plate as described as above. 4% formaldehyde was added in 0.1M PBS for 20 minutes at room temperature. The cells were washed for 5 times with PBS containing 0.1% Triton X-100 for 5 minutes per wash. 150μl blocking buffer was added into each well for 90 minutes at room temperature on a rotator. Goat anti-cathepsin B antibody (1:500 dilution) and rabbit anti-β-actin (1:2000 dilution) antibody were incubated with cells overnight at 4°C without shaking. After 5 times wash, donkey anti-goat Alexa Fluro 680 antibody (1:200 dilution) and donkey anti-rabbit Alexa Fluro 790 antibody (1:500 dilution) were added to each well for 1 hour incubating with gentle shaking at room temperature. The 96-well plate with SAHA and Cystatin C treatment was scanned to detect in both 700nm and 800nm channels using an odyssey infrared imaging system. The integrated intensity of the images was analyzed by ImageJ software (National Institutes of Health, NIH) and the signal relative values for different treatment groups were quantified as the average 800nm channel integrated intensities from duplicate wells normalized to the 700nm channel signal integrated intensity.

### LC3II fluorescence microscopy

MDA-MB-231 or MCF-7 cells were incubated with SAHA and Cystatin C in 6-well plate as indicated above. The cells were fixed with 4% formaldehyde for 10 minutes at room temperature and blocked with buffer solution containing 10% goat serum, 0.3M glycine, 1% BSA and 0.1% tween for 2 hours at room temperature. LC3II antibody solution was added to incubate with cells overnight at 4°C. A DyLight 488 fluoresence antibody (1:200 dilution) was used for an hour incubation period, and nuclei were counterstained with DAPI dye for another 10 minutes. The cells were imaged, and autophagy signals were visualized by fluorscence microscopy.

### CTSB activity assay

MDA-MB-231 or MCF-7 cells were harvested as indicated above. Cellular lysate was gathered and the activity of CTSB was performed using the colorimetric ELISA assay according to the manufacturer’s instruction. The enzymatic activity of pro-CTSB was detected by a microplate reader at 450nm.

### Human MAPK antibody array

First, approximately 2×10^7^ cells with SAHA and Cystatin C treatment were solubilized in lysis buffer. The lysates were resuspended gently at 4°C for 30 minutes and centrifuged at 14000 g for 5 minutes. Protein concentrations of the resulting lysates were measured using a BCA protein assay kit. Next, each of antibody-coated array membranes was blocked for 1 hour. 400ug of prepared cell lysates were incubated with reconstituted detection antibody cocktail at room temperature for 1 hour. Transferred the prepared sample/antibody mixtures to each well of the dish to incubate at 4°C with gentle shaking overnight. The membranes were washed with wash buffer and then incubated with 2ml streptavidin-HRP for 1 hour on a rocking platform shaker. After a final wash, membrane intensity was acquired using chemiluminescence and pixel densities can be analyzed using Gelpro Analyzer software (Media Cybernetics, Rockville, MD). Densities were measured as a percentage of the positive controls included on each membrane. After subtracting background signals and normalization to positive controls, comparisons of signal intensities among array images were used to determine relative differences in expression levels of each protein between groups.

### Data analysis

*Student*’s *t*-test was used for data analysis. Data were presented as mean ± SEM. Values for *p*< 0.05 were considered statistically significant. The model included the main effects of treatments and replicates.
